# Functional tests in patients with ischemic heart disease

**DOI:** 10.25122/jml-2019-0142

**Published:** 2022-01

**Authors:** Rodica Lucia Avram, Alexandru Cristian Nechita, Marius Nicolae Popescu, Matei Teodorescu, Liviu-Nicolae Ghilencea, Diana Turcu, Elena Lechea, Sean Maher, Gabriel Cristian Bejan, Mihai Berteanu

**Affiliations:** 1. Cardiology Department, Sf. Pantelimon Emergency Hospital, Bucharest, Romania; 2. Internal Medicine Department, Carol Davila Medicine and Pharmacy University, Bucharest, Romania; 3. Rehabilitation Department, Elias Emergency Hospital, Bucharest, Romania; 4. Cardiology Department, Elias Emergency Hospital, Bucharest, Romania; 5. Surgery Department, St. Vincent’s University Hospital, Dublin, Ireland; 6. Private Medical Practice Office Bejan Gabriel Cristian, Bucharest, Romania

**Keywords:** functional walk tests, functional capacity, ischemic heart disease, walking speed, Timed Up and Go test, WS – walking speed, TUG – Timed Up and Go, CCS – chronic coronary syndrome, STEMI – ST elevation myocardial infarction, CPET – cardiopulmonary exercise testing, IHD – ischemic heart disease, 6MWT – 6-minute walk test, HF – heart failure, IMT – intima-media thickness, MDRD – Modification of Diet in Renal Disease, CKD – chronic kidney disease, COPD – chronic obstructive pulmonary disease

## Abstract

Lately, easier and shorter tests have been used in the functional evaluation of cardiac patients. Among these, walking speed (WS) and Timed Up and Go (TUG) tests are associated with all-cause mortality, mainly cardiovascular and the rate of re-hospitalization, especially in the elderly population. We prospectively analyzed a group of 38 patients admitted to the Cardiology Clinic from Elias Hospital, Romania, with chronic coronary syndrome (CCS) (n=22) and STEMI (n=16). We assessed the patients immediately after admission and before discharge with G-WALK between the 1^st^ and 30^th^ of September 2019. Our study group had a mean age of 62.7±12.1 years. Patients with a low WS were older (69.90±12.84 *vs.* 59.90±10.32 years, p=0.02) and had a lower serum hemoglobin (12.38±1.20 *vs.* 13.72±2.07 g/dl, p=0.02). The WS significantly improved during hospitalization (p=0.03) after optimal treatment. The TUG test performed at the time of admission had a longer duration in patients with heart failure (14.05 *vs.* 10.80 sec, p=0.02) and was influenced by patients’ age (r=0.567, p=0.02), serum creatinine (r=0.409, p=0.03) and dilation of right heart chambers (r=0.399, p=0.03). WS and TUG tests can be used in patients with CCS and STEMI, and are mainly influenced by age, thus having a greater value among the elderly.

## Introduction

Worldwide, ischemic heart disease (IHD) is the most widespread cause of death, responsible for approximately 1.8 million deaths annually or 20% of deaths in European countries, with variations between them due to the different prevalence of cardiac risk factors [1]. Functional capacity represents the ability of a person to perform a variety of daily activities that require sustained aerobic metabolism.

The term frailty denotes a multidimensional syndrome characterized by increased vulnerability and reduced ability to tolerate physiological stress, including recovering from a stressor. Moreover, frailty is a predictor of developing and accelerating premature cardiac events in the general population and patients with IHD and is associated with low physical performance [2, 3]. Functional and exercise capacity and tolerance imply that the individual has performed a maximal or a sub-maximal exercise test. A patient’s level of exercise limitation is generally assessed through cardiopulmonary exercise testing (CPET). However, peak VO_2_ measurement using CPET is an expensive method that requires specialized equipment and personnel. The 6-minute walk test (6MWT) is complementary to CPET and has a proven prognostic value in patients with heart failure (HF) [4, 5] and stable coronary artery disease [6] by evaluating submaximal effort performance. Despite the frequent use of 6MWT in current practice, musculoskeletal, balance, and vascular disorders may limit the walking distance, thus making it difficult to perform in all cardiac patients. Also, the measurement of 6MWT requires a long walking path, which is not always feasible in some settings. The walking speed (WS), an easy parameter to determine, has been associated with all-cause mortality [7], mainly cardiovascular [8], and the rate of re-hospitalization [9], especially in the elderly population being suggested by some authors as the sixth vital sign [10]. The WS reflected the functional and general health status and was correlated with the risk of falling [11] and disability [12]. Few studies have evaluated the relationship between WS and cardiovascular risk factors. In the study of Elbaz *et al.*, low WS was correlated with carotid plaques and a higher IMT [13]. Also, persistent arterial hypertension was associated with lower WS and a higher decline in WS in the elderly population [14]. WS is considered a reliable indicator of vitality that integrates the cardiovascular, pulmonary, nervous, and musculoskeletal systems, besides support and balance [15]. WS and 6MWT showed similar prognostic predictive ability for all-cause mortality in older patients with cardiovascular disease [16]. The TUG test is simple to perform, reproducible, and validated for assessing balance and mobility in the elderly [17] and the risk of falling in different populations [18]. In a recent study published in 2019, Dodson *et al.* concluded that the functional mobility assessed by the TUG test is the most important predictor of the risk of readmission in patients with myocardial infarction (MI) [19]. The TUG test had a good correlation with 6MWT in patients with cardiovascular pathology, especially HF [20].

This study aimed to assess the differences between the functional mobility evaluated by the WS and the TUG test in two categories of patients with IHD: chronic coronary syndrome (CCS) and ST-segment elevation myocardial infarction (STEMI). In addition, this study aimed to identify factors that influence functional mobility and evaluate possible improvements during index hospitalization for a cardiac event following optimal therapy, medical and/or interventional.

## Methods

We prospectively analyzed a cohort of 38 patients consecutively admitted to the Cardiology Clinic of Elias Hospital in Bucharest, Romania, with stable and acute coronary syndrome between the 1^st^ and 30^th^ of September 2019.

### Study population

Two groups of patients were analyzed: the first group with CCS (n=22 patients, 57.9%) and the second group consisting of 16 patients (42.1%) with STEMI. Exclusion criteria were inability to walk for any reason, reduced vision or blindness, previously documented changes in gait (Parkinson’s disease, persistent changes secondary to a stroke), severe cognitive disorder, and patient’s refusal.

### Trial design

After inclusion in the study, we evaluated patients clinically and biologically (current symptoms, blood pressure and heart rate, standard laboratory samples). In addition, we collected demographic data as well as associated comorbidities and home drug therapy. Patients underwent an ECG, echocardiography, and functional exploration using the G-walk device. At the time of inclusion in the study, patients were stable, and consent was obtained from the attending physician. Patients performed another G-walk test at discharge, and any adverse events were monitored during hospitalization.

### Functional mobility evaluation

We assessed the functional mobility with a portable wireless inertial sensing device (G-walk, BTS Bioengineering, Quincy, MA, USA). G-walk is a gait analysis system that measures the subject’s center of mass using a wireless triaxial accelerometer. The parameters calculated from the data were transferred wirelessly via Bluetooth for analysis by BTS G-studio software. When performing the TUG test, the patients were asked to get up from a chair with a height of 46 cm, walk 3 meters, and return to the chair at their normal walking pace. The time was recorded in seconds. Patients first performed a non-timed TUG test to familiarize themselves with the commands. TUG test results were divided into preserved mobility (TUG <15 s), mild impairment (TUG 15–25 s), moderate impairment (TUG>25 s), and severe impairment (unable to complete assessment). The WS was measured with the same device using a semi-elastic belt with the inertial sensor attached at the lower lumbar level (centered on the L4-L5 intervertebral disc) in an area set up in the hospital corridor by asking the patient to walk 2x6m with a turn. Patients were allowed to choose which way to turn, and they were asked to wear shoes. Adhesive tape strips on the floor indicated the start point. A standing start for the test was chosen for the test since a rolling start would mean that more space would be needed. The patient was asked to walk at a comfortable pace. Vocal instructions were provided (e.g., “walk at a comfortable pace”). The patients performed a non-timed test to familiarize themselves with the commands. The WS was recorded as the average instantaneous speed within the gait cycle. Low WS was defined as <0.8 m/s. Functional tests were performed by a physician in the study team.

### Biological and ultrasound parameters

The standard biological samples analyzed were those taken at admission, except for troponin I, for which we noted the maximum measured value. Creatinine clearance was calculated based on the Modification of Diet in Renal Disease (MDRD) formula. In addition, we performed a cardiac ultrasound on admission.

### Statistical analysis

Statistical analysis was performed using SPSS 20 (Statistical Package for Social Sciences, IBM, Armonk, New York, USA) and MedCalc (MedCalc Statistical Software, Ostend, Belgium). We used numeric and categorical parameters. Bartlett’s test for homogeneity was used to determine if variances are equal between compared variables. Homogeneous numerical variables were expressed as means±standard deviation, and comparisons were made with the ANOVA parametric test. Inhomogeneous numerical variables were expressed as the median and interquartile range (IQR: 25%–75% percentiles) and compared using the Mann-Whitney/Wilcoxon two-sample test. Categorical parameters were expressed as percentages, and associations were assessed with the Chi-squared corrected test and expressed as odds ratios (OR) with 95% confidence intervals (95%CI). Using the Enter method, multivariate logistic regression models were utilized to identify independent predictors of outcome. All p-values were two-sided, and values less than 0.05 were considered statistically significant.

## Results

Our study group consisted of 38 patients with a mean age of 62.7±12.1 years. 57.9% of patients were admitted with CCS diagnosis and the rest with STEMI. The patients’ clinical characteristics are listed in Table 1. The median duration of hospitalization of the whole group was 4 days (IQR: 3.25), longer in patients admitted with the diagnosis of STEMI compared with CCS [5 (IQR: 4) *vs.* 3 (IQR: 3), p=0.009]. The average WS (m/s) on the admission of the whole group was 0.97±0.2. On initial evaluation, performed close to the time of admission, the WS (m/s) did not differ statistically between the two groups of patients analyzed with CCS and STEMI (0.96 *vs.* 1, p=0.64).

**Table 1.** Patient characteristics.

	**All (n=38)**	**CCS (n=22, 57.9%)**	**STEMI (n=16, 42.1%)**	**p-value**
**General Characteristics**				
Age (years)±SD	62.7±12.1	63.7±11.5	61.2±13.2	0.54
Sex (male), n (%)	24 (63.2%)	13 (59.1%)	11 (68.7%)	0.78
**Risk factors, n (%)**				
Diabetes mellitus	16 (42.1%)	10 (45.5%)	6 (37.5%)	0.76
Hypertension	30 (78.9%)	19 (86.4%)	11 (68.7%)	0.25
Dyslipidemia	27 (71.1%)	20 (90.9%)	7 (43.7%)	0.01
Obesity	12 (31.6%)	6 (27.3%)	6 (37.5%)	0.71
Ever Smoker	24 (63.2%)	12 (54.5%)	12 (75%)	0.30
**Cardiovascular disease, n (%)**				
Prior myocardial infarction	7 (18.4%)	6 (27.3%)	1 (6.2%)	0.67
Peripheral artery disease	8 (21.1%)	6 (27.3%)	2 (12.5%)	0.68
Heart failure	16 (42.1%)	14 (63.6%)	2 (12.5%)	0.18
Stroke	3 (7.9%)	3 (13.6%)	0	NA
**Other chronic conditions, n (%)**				
COPD	2 (5.3%)	2 (9.1%)	0	NA
Depression	5 (13.2%)	5 (22.7%)	2 (12.5%)	0.77
Chronic renal disease	2 (5.3%)	2 (9.1%)	0	NA
**Angiography, n (%)**				
Normal epicardial arteries	4 (10.5%)	4(18.1%)	0	NA
Cardiac catheterization only	13 (34.2%)	11 (50%)	2 (12.5%)	0.3
PCI	21 (55.2%)	7 (31.8%)	14 (87.5%)	0.01
Residual lesions	21 (55.2%)	11(50%)	10 (62.5%)	0.57

COPD – chronic obstructive pulmonary disease; ACE – angiotensin-converting enzyme; ARB – angiotensin receptor blockers; PCI – percutaneous coronary intervention; SD – standard deviations.

No significant correlation was observed between WS and cardiovascular risk factors such as diabetes mellitus, arterial hypertension, dyslipidemia, obesity, and BMI. There were no gender differences in terms of WS. There were no differences in WS between patients with or without the peripheral arterial disease (PAD), pre-existing IHD, chronic kidney disease (CKD), stroke, chronic obstructive pulmonary disease (COPD), and heart failure (HF) of the associated comorbidities analyzed. We did not find any significant correlation between WS and the analyzed cardiac ultrasound parameters.

Patients with a low walking speed were older (69.9±12.84 years *vs.* 59.9±10.32 years, p=0.03) and had lower serum hemoglobin (12.38±1.20 g/dl *vs.* 13.72±2.07 g/dl, p=0.02). When we evaluated the correlation of age and WS differentiated on the two groups, we found that this association reached statistical significance only in the CCS patients (72.66±13.8 years *vs.* 60.42±9.04 years, p=0.02). The WS significantly improved during hospitalization (p=0.035) after optimal treatment administrated in the cardiology department (interventional or medical). The mean value of the TUG test (seconds) performed on the whole group was 12.34±3.9. The TUG test (seconds) completed at admission was longer in patients with CCS than STEMI patients (13.44 *vs.* 9.6, p=0.002). TUG test was longer in patients with previously documented IHD (13.2 *vs.* 9.6, p=0.02), stroke (20.5 *vs.* 11.3, p<0.001), CKD (18.8 *vs.* 11.8, p=0.01), and HF (14.1 *vs.* 10.8, p=0.02) compared to those with no previous history of these comorbidities. The TUG test value was strongly influenced by patients’ age (r=0.567, p=0.02), and moderately influenced by serum creatinine level (mg/dl) (r=0.409, p=0.03) and creatinine clearance (ml/min/1.73 m^2^) (r=0.398, p=0.04). Among the echocardiographic parameters, dilation of the right heart chambers was associated with an increased value of the TUG test (r=0.399, p=0.03). 21.43% of patients had a TUG test value greater than 15 seconds, a value that is correlated with impaired mobility. We present the TUG test and OR for risk factors in Table 2, and the outcome characteristics are presented in Table 3. There were no adverse events during the functional mobility assessment.

**Table 2.** Timed Up and Go (functional capacity) and OR for risk factors.

	**OR**	**95%CI**	**p-value**
**STEMI diagnosis on admission**	1.85	0.27–12.76	0.53
**Diabetes mellitus**	6.6	1.23–35.44	0.02
**HTN**	2	0.15–26.73	0.59
**Dyslipidemia**	3.81	0.68–21.42	0.11
**History of medication before admission**	1.28	1.03–1.58	0.25

HTN – Hypertension; STEMI – ST-elevation myocardial infarction; OR – odds ratio.

**Table 3.** Outcome characteristics.

	**All patients (n=38)**	**CCS (n=22, 57.9%)**	**STEMI (n=16, 42.1%)**	**p-value**
**Duration of hospitalization (days), median (IQR)**	4 (3.25)	3 (3)	5 (4)	0.009
**PCI, n (%)**	21 (55.2%)	7 (31.8%)	14 (87.5%)	0.01
**Walking speed on admission (m/s), mean±SD**	0.97±0.2	0.96±0.21	1±0.18	0.63
**Walking speed at discharge (m/s), mean±SD**	0.99±0.28	0.99±0.24	0.99±0.32	0.96
**Timed up and Go on admission (s), mean±SD**	12.34±3.9	13.44±4	9.6±1.81	0.002
**Timed up and Go at discharge (s), mean±SD**	13.17±5.06	13.59±4.94	12.68±5.31	0.61

PCI – percutaneous coronary intervention; SD – standard deviations; IQR – interquartile range.

## Discussion

Measuring the WS over a short distance is an easier method to implement in clinical practice and an objective parameter that can be evaluated repeatedly. Increased HDL cholesterol was not associated with better motor performance in our study population, although this was shown in a previous report by Volpato *et al.* [21]. Although diabetes has been associated in some cases with mild symptoms of parkinsonism [22] or decreased cerebral vasoreactivity [23], no difference in WS was observed in our group of patients. In our study, WS was not significantly correlated with cardiovascular risk factors analyzed as opposed to available data from the literature. A possible explanation could be that our patients were approximately 10 years younger compared to other studies (73.7 years (21) and 73.3±4.7 years (13)). 57.9% of the patients were younger than 65 years at the time of admission. A significant improvement in WS was observed during the hospitalization period after optimal individualized treatment for each patient, a parameter that was associated in other studies with a favorable long-term prognosis [24]. There are several explanations for this correlation, such as the existence of a physiological reserve specific to each patient [25] or a different ability to recover after a major medical event [26] together with the applied medical interventions.

Although the WS can change over time [27], the most effective therapy for improving the prognosis of patients with cardiovascular disease and low WS, a marker of frailty, is relatively weakly established. Despite advances in pharmacotherapy and even innovative cardiovascular regenerative medicine, with major emphasis on the quality and quantities of regenerative products [28], none of these interventions have proven to fully meet these patient’s needs. Of these therapies, physical exercise is the most promising and studied intervention [29]. Considering that low WS was correlated with older age in our group, we believe all efforts should be made to include this set of patients in cardiac rehabilitation programs. Furthermore, as low WS almost doubled the risk of readmissions and death in patients with MI [30], we recommend measuring it systematically in this population and closely monitoring patients with low values.

The TUG test had a longer duration in patients with IHD and associated HF, similar to the results of Hwang *et al.* [20]. Patients with pre-existing IHD had a longer duration of the TUG test than those without heart disease, as also demonstrated by Albarrati *et al.* in a post-CABG versus control population [31]. In previous studies, the value of the TUG test was related to age in a similar manner to the results of the present research. In addition, the TUG test was correlated in our study with serum creatinine and pre-existing CKD. Similar to other studies in which CKD was associated with higher odds of self-reported incident motor disability [32], we found increased creatinine level as a predictor of poor physical function or frailty in our patients. The TUG score and WS are considered surrogate markers for frailty [33], a proven significant risk factor for adverse events in patients with acute MI [34].

The cohort is further followed, and longitudinal analysis will be carried out in the future to show if improvement in WS during hospitalization is associated with a prognostic significance. Given the rising importance of chronic debilitating diseases, many of which are cardiovascular in origin, the evaluation of physical abilities is an essential factor in routine clinical practice of the primary care providers, cardiologists and rehabilitation specialists. Functional capacity evaluation is a multi-domain process in which aerobic ability, strength, balance, physical frailty, and cognition intertwine. Although the TUG test and WS are not a measure of aerobic capacity, they are reliable and robust markers of frailty and have a good correlation with measures of exercise capacity in terms of prognosis. These functional tests can be used as simple assessment tools, especially when CPET or 6MWT are unavailable or cannot be performed. This study was conducted with a relatively small number of patients at a single center. As we only assessed effort capacity during the hospitalization period, we could not evaluate the long-term predictive significance. Moreover, we did not evaluate the cognitive function and quality of life of our patients.

## Conclusions

Our study showed that WS and the TUG test can be used without harm in patients with CCS and STEMI and that their outcomes are mainly influenced by age. Subsequently, these tests thus have a greater utility in the elderly population with ischemic heart disease. WS improved during hospitalization after optimal individualized therapy. However, further research should assess this relationship in order to establish the predictive significance.

## Acknowledgements

### Conflict of interest

The authors declare no conflict of interest.

### Ethics approval

The study was approved by the Ethics Committee of the Emergency Hospital Sf. Pantelimon (approval number 49/2018).

### Consent to participate

Written informed consent was obtained from the participants in the study.

### Authorship

RLA contributed to conceptualizing, data collection, data analysis and writing the original draft. ACN and MB contributed to conceptualizing, MNP contributed to the methodology and data collection. MT and EL contributed to data collection. LNG and GCB contributed to data analysis. DT contributed to data collection. SM. Maher contributed to writing the original draft.
